# Pragmatic non-inferiority Randomised trial Investigating Needle aspiration versus ChEst drain for Secondary Spontaneous Pneumothorax (the PRINCE-SSP study): study protocol for a randomised non-inferiority trial

**DOI:** 10.1136/bmjopen-2024-093700

**Published:** 2024-12-26

**Authors:** Steven Walker, Ellie Taylor, Amy McAndrew, Edward Carlton, Ella Chaudhuri, Pooja Ghorpade, Charlotte Goodwin, James Connors, Heather Cook, Ramon Luengo-Fernandez, Siobhan Creanor, Nick A Maskell

**Affiliations:** 1 Academic Respiratory Unit, University of Bristol, Bristol, UK; 2 North Bristol NHS Trust, Westbury on Trym, Bristol, UK; 3 Exeter Clinical Trials Unit, University of Exeter, Exeter, UK; 4 University of Bristol, Bristol, UK; 5 Health Economics Research Centre, University of Oxford, Oxford, UK

**Keywords:** Randomized Controlled Trial, Adult thoracic medicine, ACCIDENT & EMERGENCY MEDICINE

## Abstract

**ABSTRACT:**

**Introduction:**

Secondary spontaneous pneumothorax (SSP) is a medical emergency where the lung collapses in the presence of underlying chronic lung disease. Current international clinical guidelines advise intercostal drain (ICD) insertion for SSP. However, in a previous small study needle aspiration (NA) has been shown to reduce length of hospital stay (LOHS) and reduce complications. We are evaluating the clinical and cost-effectiveness of an initial NA approach to the management of patients with SSP in the United Kingdom.

**Methods and analysis:**

The PRINCE-SSP (Pragmatic non-inferiority Randomised trial Investigating Needle aspiration vs ChEst drain for Secondary Spontaneous Pneumothorax) trial is a pragmatic, multicentre, open-label, parallel, two-group, randomised, non-inferiority trial to establish whether NA for SSP is non-inferior in terms of LOHS compared with ICD. We aim to recruit 110 patients from at least 15 UK NHS hospitals, over 18 months. Participants allocated to the intervention (NA) group will have an NA inserted at the presentation. Those allocated to the comparator (usual care) group will have an ICD inserted. Participants are followed up for 30 days. The primary outcome measure is initial LOHS, up to day 30 postrandomisation. Secondary outcomes include (but are not limited to) total LOHS including readmissions, complications, cost-effectiveness and patient-reported quality of life.

**Ethics and dissemination:**

This trial received Health Research Authority (HRA) approval from Wales Research Ethics Committee seven ethics committee (23/WA/0085). Results will be submitted for publication in a peer-reviewed journal. A plain English summary of the trial results will be prepared and disseminated with the help of our patient advisory group, including via social media, and provided to trial participants via post or email according to their preference.

**Trial registration number:**

ISRCTN12644940.

**STRENGTHS AND LIMITATIONS OF THIS STUDY**

- The trial uses techniques already used in other types of pneumothoraces, with anticipated rapid translation of results into routine clinical management of secondary spontaneous pneumothorax (SSP), and incorporation into national and international guidelines.
- Patients with absent or diminished capacity can be recruited, to expand generalisability to critically unwell patients who are unable to consent at presentation.
- The trial involves an economic evaluation to determine the cost-effectiveness of needle aspiration versus intercostal drain of SSP.
- Blinding to treatment allocation is not possible for clinicians or participants.

## Introduction

 Secondary spontaneous pneumothorax (SSP) is a medical emergency where the lung collapses in the presence of underlying chronic lung disease, such as chronic obstructive pulmonary disease (COPD). It is the most common cause of spontaneous pneumothorax, with 6000 patients per year admitted to hospitals with SSP in England.^1^ SSP causes significant breathlessness and pain and results in higher morbidity, mortality and longer hospital admissions than patients with pneumothoraces and no underlying lung disease (‘primary spontaneous pneumothorax’ (PSP)).^2^ SSP typically occurs in the elderly and people with comorbidities, with an average age of 60–65.^3^

Current management of SSP is the insertion of an intercostal drain (ICD) to drain the pneumothorax.^4 5^ The drain requires a chest wall incision, guidewire insertion and dilation of the entry tract. It is then stitched into place and attached to a drainage system (underwater seal drain) for at least 24 hours and requires hospital admission for all patients, with an average length of stay of 10 days.^6 7^ An alternative method is needle aspiration (NA), where a small-bore cannula is inserted into the chest and the air is manually aspirated with a syringe, before removing the cannula as soon as all the air is removed. This takes approximately 20 min and is a one-off procedure. NA is a simpler procedure than a chest drain, less painful for patients, less time-consuming for clinicians, associated with fewer complications and can lead to same-day discharge.^8^

The use of NA in PSPs has been extensively studied, with 11 reported randomised controlled trials comparing NA to ICD.^9^,^20^ These studies have shown NA to be an effective method for managing PSP, with a meta-analysis demonstrating a significant mean reduction in length of hospital stay (LOHS) of 2.55 days (95% CI: 2.24 to 2.87) in favour of NA.^4^ Based on these studies, the European Respiratory Society 2024 advises NA as a first-line option in patients with PSP,^5^ and all UK emergency, acute and respiratory physicians are trained in NA and perform it regularly.^6^

While the less invasive, immediate drainage technique offered by NA appears also desirable for patients with underlying lung disease (ie, patients with SSP), concerns about the efficacy of NA in SSP were raised from two small case series reports.^21 22^ This has been challenged by the findings of a recent randomised study,^17^ which recruited 48 patients with SSP as part of a larger cohort of patients, which also included 79 patients with PSP.^17^ In the SSP subgroup, median (IQR) LOHS in the NA arm was 2.5 days (1.2–7.8) versus 5.5 days (3.6–9.2) in the chest drain arm (p=0.049). NA was also associated with higher rates of immediate success in the SSP subgroup: 59% for NA compared with 23% in the chest drain group (p=0.011). Importantly, complication rates across all patients were much higher in the chest drain arm than NA (15 serious complications vs none).^17^

### Aims and objectives

The Pragmatic non-inferiority Randomised trial Investigating Needle aspiration versus ChEst drain for Secondary Spontaneous Pneumothorax (PRINCE-SSP) trial will test whether NA is no worse than ICD in patients with SSP with respect to initial LOHS while demonstrating benefits in other important patient-reported, clinical and cost-effectiveness outcomes.

Specific objectives are:

- To test whether NA is non-inferior to chest drain with respect to initial LOHS during 30 days from randomisation
- If NA is non-inferior to usual care, to assess whether NA is superior to chest drain with respect to initial LOHS
- To estimate between-group differences in patient-reported outcomes
- To estimate between-group differences in clinical outcomes
- To estimate between-group differences in healthcare resource usage and costs
- To assess the cost-effectiveness of NA versus ICD

## Methods and analysis

### Trial design

The PRINCE-SSP trial is a pragmatic, multicentre, open-label, parallel, two-group, randomised, non-inferiority trial.

### Setting

Participants will be recruited from 20 hospital sites across England and Wales (see table 1). Each site must have an emergency department (ED) or acute medical unit (AMU). All sites will treat and manage continued care for trial participants as per usual NHS care.

**Table 1:** Recruiting **h**ospitals

Addenbrooke’s Hospital, Cambridge	Royal London Hospital, London
Blackpool Victoria Hospital, Blackpool	Royal United Hospital, Bath
Canterbury Hospital, East Kent	Sheffield Teaching Hospitals NHS Foundation Trust Sheffield
Glenfield Hospital, Leicester	Southmead Hospital, Bristol
Hull Royal Infirmary, Hull	St James’s Hospital, Leeds
Macclesfield Hospital, East Cheshire	Torbay and South Devon NHS Foundation Trust. Torbay
Musgrove Park, Taunton	University College London Hospitals NHS Foundation Trust, London
Norfolk and Norwich University Hospitals Trust, Norfolk	University Hospital of Wales, Cardiff
Oxford University Hospital NHS Foundation Trust, Oxford	University Hospitals Plymouth NHS Trust, Plymouth
Princess Alexandra Hospital, Harlow	Wythenshawe Hospital, Manchester

### Trial population

**Figure 1:**
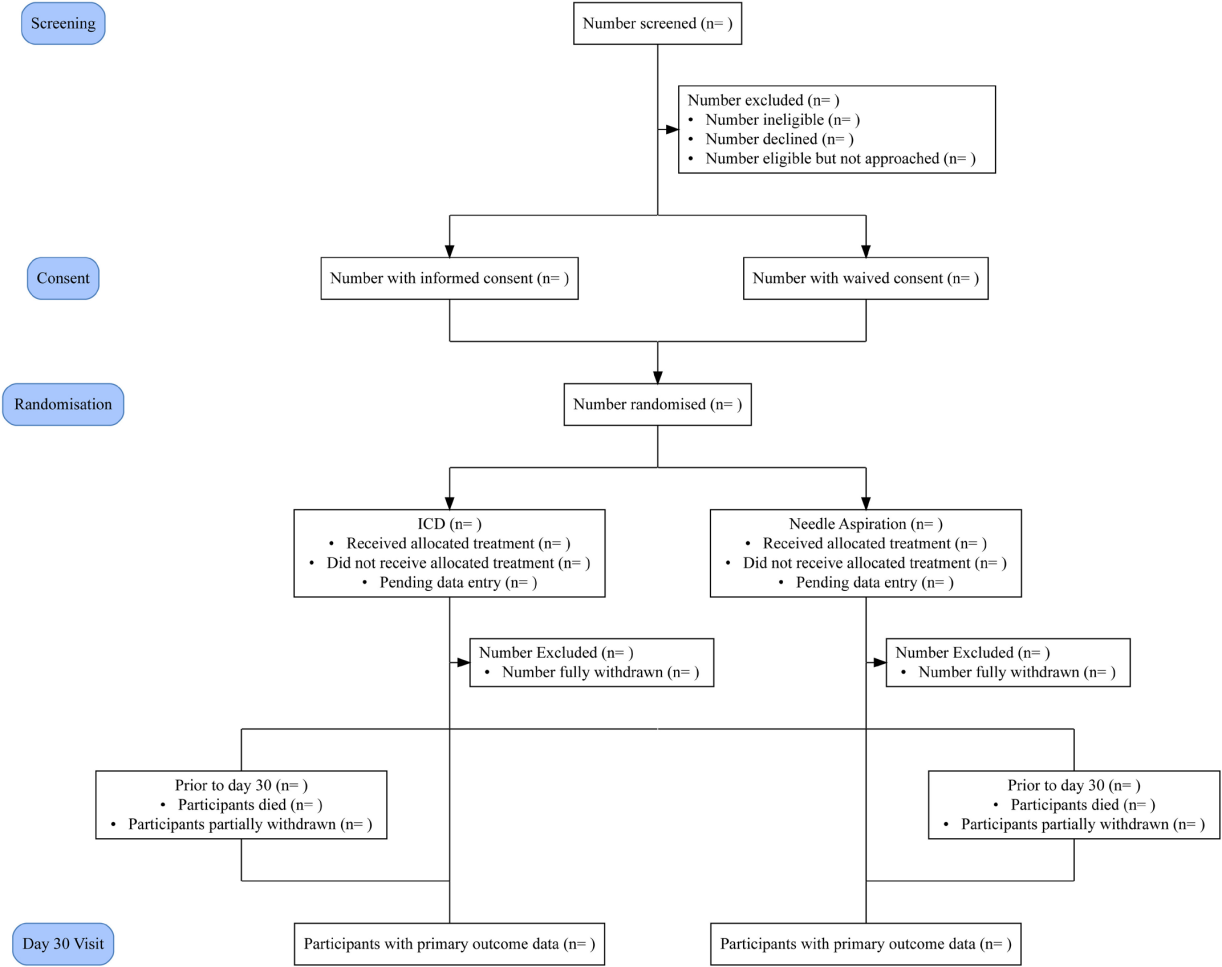
Trial schema illustrating the pathway for PRINCE-SSP (Pragmatic non-inferiority Randomised trial Investigating Needle aspiration versus ChEst drain for Secondary Spontaneous Pneumothorax) participants. ICD, intercostal drain.

The target population is patients with a diagnosis of SSP, presenting to emergency, acute medical and respiratory departments at a UK hospital. Patients may be eligible if they are at least 18 years of age, and the treating clinician believes that the pneumothorax is of sufficient size and symptoms for the treating physician to consider intervention with ICD. SSP is defined in this trial as defined as a pneumothorax occurring in a patient with known underlying lung disease, or in a patient at least 50 years of age with significant smoking history or in a patient with suspected underlying lung disease with radiology confirming structural lung disease. Exclusion criteria are bilateral pneumothorax, traumatic or iatrogenic pneumothorax, clinical concerns of tension pneumothorax, younger than <18 years of age or known pregnancy (figure 1).

### Primary outcome

Initial LOHS from randomisation, up to 30 days after randomisation. This will be calculated to the nearest hour. To enable supporting analyses of the primary outcome, every attempt will be made to collect the date of discharge if beyond day 30, to calculate the initial LOHS for all participants.

### Secondary outcomes

The secondary outcomes will capture the possible advantages of the intervention for patients in terms of reduced pain, complications and improved health-related quality of life in the short term, as well as inform a formal cost-effectiveness analysis.

Secondary outcomes are as follows: (1) total number of days in hospital up to 30 days after randomisation, including readmissions; (2) immediate treatment success: for NA, success defined by persistent adequate response following the aspirations; for ICD, success is defined by adequate response with removal of drain within 72 hours of drain insertion; (3) hospital readmission within the first 30 days after randomisation; (4) pain (assessed using Numerical Rating Scale) and breathlessness (MRC Dyspnoea Scale^23^), measured postintervention (within 24 hours), daily over first 5 days and at day 30 postrandomisation; (5) patient-reported health status (EuroQol 5 dimension 5 level [EQ-5D-5L] and short form 36 [SF-36] questionnaire) measured postintervention (within 24 hours), day 5 and day 30 postrandomisation; (6) Clinical Frailty Scale^24^ measured postintervention and day 30 postrandomisation; (7) X-ray resolution (taken during routine care visit) at day 30 postrandomisation; (8) complications (yes/no for relevant predefined events) up to day 30 postrandomisation; (9) total number of subsequent pleural procedures (a) within first 24 hours and (b) up to day 30 postrandomisation; (10) mortality within the first 30 days after randomisation; (11) hospital resource use up to day 30 postrandomisation, including emergency, admitted, critical and outpatient care; (12a) cost-effectiveness: the incremental cost per each of the following secondary outcomes: pain, breathlessness and number of complications prevented when NA is compared with chest drain over 30 days; (12b) cost-utility: the incremental cost per quality adjusted life year (QALY) (gained) when NA is compared with ICD over 30 days.

### Sample size

Three relevant randomised studies have compared NA and ICD over the last decade at the time of protocol drafting^17^,^19^; two included patients with only PSP, and one included patients with both PSP and SSP. Synthesis of LOHS in these studies yields a mean between-group difference of −2.5 days (95% CI: −3.5 to −1.6) in favour of NA and pooled SD of 3.8 days. The median LOHS in the SSP subgroup (n=48) was 2.5 days in the NA arm versus 5.5 days in the chest drain arm, with a pooled SD of 4.7.^17^

The study aims to recruit 110 participants and, allowing for up to 5% mortality rate within 30 days and nominal loss to follow-up/incomplete routine LOHS data, expects to collect LOHS for approximately 50 participants per allocated group. 100 participants allocated 1:1 to NA and chest drain groups, and assuming a true between-group difference of −2 days (in favour of NA) and SD of 4.6 days, provides 90% power to declare non-inferiority at the one-sided 2.5% significance level, using a non-inferiority margin of +1 day. If non-inferiority is declared, superiority will be assessed: this sample size will allow detection of a between-group difference in LOHS of 3 days with 90% power, or 2.6 days with 80% power, at the two-sided 5% significance level. With this sample size, the probability of declaring superiority (Bayesian assurance), assuming a prior informed by the synthesised mean between-group difference of −2.5 days, is 75%.

### Patient approach, recruitment and randomisation

Potentially eligible participants will be identified by clinical and research teams in the emergency, acute medical and respiratory departments. All potential participants will be given a patient information sheet (PIS) describing the study. The timing at which patients receive the PIS will vary, depending on their capacity at time of identification. Patients will require a chest X-ray to confirm presence of pneumothorax.

Following a confirmed diagnosis of SSP and eligibility assessment by the usual care team, eligible patients undergo a capacity assessment from a suitably trained clinical staff member. If at this point, the patient has capacity, they are approached in the ED/AMU for their consent to take part in the study.

When patients are judged to be unable to provide informed consent for themselves at the time a decision or action needs to be taken, they can be automatically enrolled in the trial under deferred consent. The patient will then receive the randomly allocated trial intervention. If patients regain capacity within 72 hours postrandomisation, they are approached and asked to provide consent to continue in the trial. If patients do not regain capacity within 72 hours of randomisation, a member of the research team seeks advice from a personal consultee or, if unavailable, a nominated consultee.

Participants are randomised immediately after SSP has been diagnosed and informed consent is provided or waiver of consent is applied. Participants are allocated in a 1:1 ratio to either NA (intervention group) or intercostal drain (comparator group) using a secure, internet-based system, RedCap Academic, designed and maintained by Exeter CTU. Randomisation uses a minimisation algorithm with a random element, with two minimisation factors: (1) first/recurrent pneumothorax and (2) underlying lung disease (COPD vs other).

### Trial interventions

In the intervention (NA) group, the treating physician will use a small-bore catheter connected to a three-way valve, a 60 mL syringe and a drainage bag. The procedure will be terminated when no more air can be aspirated, or if the amount of aspirated air exceeds approximately 2.5 L. Shortly after, a chest X-ray will be performed to assess the change in pneumothorax size. Decisions regarding further intervention should be guided by the degree of the participant’s symptoms and physiology. If the participant remains symptomatic (reporting ongoing significant breathlessness) or physiologically unstable and pneumothorax is ≥2 cm (chest wall to lung edge) then the chest X-ray should be used to determine if an ICD is feasible and to guide insertion site. If drain insertion is feasible, a small-bore ICD (≤14F) should be inserted with the Seldinger technique and attached to an underwater seal bottle. This will count as a subsequent pleural procedure.

In the comparator (usual care) group, the treating physician will use a small-bore ICD (≤14F) with the Seldinger technique. The ICD should be connected to a drainage system (the underwater seal bottle). A chest X-ray will be repeated following cessation of bubbling in the water seal in the chest drainage system. Once the chest X-ray demonstrates full lung re-expansion and cessation of bubbling in the water seal, the chest drain can be removed.

### Data collection

Due to the emergency setting of the trial, the minimum essential data will be collected prior to randomisation, which will allow randomisation to be performed quickly and prevent delay in patient treatment. Clinical data will be collected at baseline and at day 30 by participating site team members onto case report forms and participant-completed questionnaires. These are entered into a REDCap^25^ database for data cleaning and analysis. Access to the database is via a secure password-protected web interface. It is acknowledged that it may not be feasible, or appropriate, to complete participant-reported outcome measures (PROMs) straight away (eg, the participant is too symptomatic or does not have capacity prior to treatment starting for their SSP). The first PROMs will be collected as soon as feasible following randomisation (within 24 hours of randomisation) and after treatment delivery, to ensure that urgent treatment is not delayed. Participants will be asked to complete the paper questionnaire according to how they feel at the time of completion, rather than retrospectively. The questionnaire may be completed with the assistance of a researcher/member of the clinical team (or person with caring responsibility, for example, family member, if/where feasible). If the participant does not have capacity, then a consultee can be asked to complete the resource use questions but no other questionnaires. PROMs will then be collected daily for the first 5 days after randomisation and at day 30.

### Statistical analysis

Data obtained will be analysed according to the intention to treat principle and reported according to the CONSORT (Consolidated Standards of Reporting Trials) guidelines.^26^ The primary analysis will compare initial LOHS, up to day 30, between allocated groups using linear mixed effects modelling, adjusting for minimisation factors and other prespecified prognostic factors, and including site as a random effect, with bootstrapping considered as necessary to handle skewed data. Participants who have died during the trial will be excluded from the primary analysis but included in all further analysis of the primary outcome data. To ensure that this is a reasonable approach, a worst/best and best/worst imputation of the missing initial LOHS data due to death will be undertaken (where best and worst are defined as ±2 SD from the mean).^27^ The between-group mean difference in initial LOHS will be presented alongside the adjusted two-sided 95% CI. If the upper limit of the one-sided 97.5% CI lies below 1 day, non-inferiority will be declared and then superiority will be assessed against the null hypothesis of no between-group difference. All secondary analyses of the primary outcome will be under the non-inferiority framework in the first instance, and subsequently for superiority if non-inferiority is demonstrated. Secondary outcomes will be assessed only for superiority.

### Health economic analysis

Information on days in hospital (including patients’ hospital pathway and specialties in which they were admitted to) and outpatient and emergency care visits will be obtained from a review of patients’ hospital electronic patient records within each participating NHS trust. A micro-costing study will be performed to assess the costs of performing each of the trial’s procedures, with the following being measured: time to conduct the intervention, staff (and grade) involved, number of disposables used, investigations and medications used. Hospital stays and outpatient and emergency care contacts will be valued using NHS Reference costs. At each trial follow-up (including baseline), the score for each of the eight dimensions of the SF-36 will be coded. QALYs will then be estimated using SF-6D and EQ-5D utilities. A within-trial cost-utility analysis will explore the incremental cost per QALY gained by NA compared with chest drain over 30 days, where the majority of the costs associated with pneumothorax are incurred.^28^

### Safety

Participant safety will be monitored by the Trial Management Group, sponsor and oversight committees (Trial Steering Committee and Data Monitoring Committee). The trial protocol contains a list of events that can be expected in this patient population.

All serious adverse events (SAEs) between randomisation and day 30 (+7 day window) will be collected. SAEs that are both related to the trial (ie, resulted from NA or ICD management or administration of a research procedure) and unexpected (ie, not listed in the protocol as expected) are classed as ‘related unexpected serious adverse events’ and will be subject to expedited reporting to the Sponsor and Research Ethics Committee (REC).

### Patient and public involvement

A patient and public involvement (PPI) group made up of PPI co-applicants/members and supplemented through networking and outreach work meet as needed throughout the duration of the trial to ensure an iterative and responsive PPI strategy. The PPI group have focused their efforts on providing feedback on trial documents and will assist in the dissemination of trial results.

### Risk of bias

Allocation to intervention or control is at random using a remote web-based system to ensure allocation concealment. Due to the nature of the intervention and usual care treatments, clinicians and participants cannot be blinded, with the further potential need for clinical decisions made by treating clinicians. The primary outcome (initial LOHS) is an objective measure, calculated from the time of randomisation and routine hospital data. Reviewers of X-ray resolution and complications will be blinded to treatment allocation. Redacted X-rays will be emailed from participating sites to North Bristol NHS Trust via NHS email to be assessed by a blinded clinician who is not associated with the study. The trial statistician will be blinded while drafting the statistical analysis plan and will become unblinded after the statistical analysis plan is approved by an independent statistician. The senior trial statistician will be unblinded throughout the trial.

### Trial management and oversight

The cochief investigators take overall responsibility for the trial. Exeter CTU (ExeCTU), a UK Clinical Research Collaboration registered clinical trials unit is responsible for the preparation of trial documents, site initiation visits and training, day-to-day running of the trial and monitoring of centres. The Trial Management Group is overseeing the trial and meets monthly to review progress. The Trial Steering Committee meets biannually to review conduct and progress, and the Data Monitoring Committee meets at least annually to review data completion and safety.

#### Trial sponsor

The trial sponsor is North Bristol NHS Trust, which oversees the trial and has ultimate responsibility for any decision about its continuation.

North Bristol NHS Trust

Southmead Hospital

Bristol

BS10 5NB

Email: researchsponsor@nbt.nhs.uk

Phone: 0117 414 9330

### Changes to trial protocol

The trial protocol has been updated in response to comments from the ethics committee. A further update occurred to correct errors within the consent process diagram in order for it to match with the text within the protocol and to remove the option of e-consent. The latest protocol update was to amend the deferred consent option to allow one doctor and one healthcare professional to consent participants who lack capacity. The SAE section was also updated to clarify reporting procedures in line with the HRA guidance. Changes have been made to the day 30 X-ray review process to allow sites to upload images to the database.

### Data statement

Data are stored on anonymised research data and outputs in the University of Exeter’s Open Research Exeter repository (https://ore.exeter.ac.uk/repository/) in order to facilitate open access to, and the impact of, our research. All future research proposals must obtain the appropriate ethical and regulatory approvals.

## Discussion

This trial is a pragmatic multicentre randomized controlled trial (RCT) aiming to establish whether NA for treating SSP is non-inferior to ICD insertion in terms of clinical and cost-effectiveness.

NA is a quicker, less invasive approach compared with ICD and may facilitate shorter hospital stays. Should an NA prove non-inferior to invasive in SSP, this is likely to lead to widespread changes in practice and prevent avoidable harm and long hospital stays from chest drain insertion.

We recognise recruitment of patient with SSP can be challenging. Patients with SSP presenting to the ED are often very symptomatic and unwell. They are typically composed of older patients from underserved populations. The only published RCT on initial management of SSP did not recruit to target.^29^ There have only been three other RCTs exclusively examining management for SSP,^30^,^32^ and each of these trials was in patients after initial treatment for SSP had been completed. The following aspects have been considered in order to ensure the trial can be successfully delivered.

### Recruitment of acutely unwell patients

The target population, identified in the emergency setting by the usual care team, poses challenges in terms of information provision and informed consent: patients will potentially be in significant pain/respiratory distress at the time of presentation, maybe hypoxic and may have diminished or absent capacity. To maximise generalisability and recruitment, the study protocol allows a waiver of consent for patients with diminished capacity under the provision of the Mental Capacity Act 2005, and approach such individuals for consent to continue once they have regained capacity.

### Out-of-hours recruitment

Enrolling participants in trials out of routine working hours in the ED, AMU and respiratory wards is challenging. Most hospitals do not have research staff working out of hours, resulting in less capacity to recruit for trials. Initial screening in the PRINCE-SSP trial demonstrated approximately 50% of potential participants were not approached due to ‘lack of staff availability’. Efforts to address this include incentivisation for trainees and allied health professionals (including authorship for high recruitment rates) by the trial team, use of National Institute for Health and Care Research associate primary investigator scheme, increasing accessibility of training requirements and use of national trainee networks such as Trainee Emergency Research Network to promote trainee engagement.

### Trial status

Recruitment began in August 2023 and is due to finish in April 2025. The PRINCE-SSP trial is currently recruiting at 19 UK organisations.

### Ethics and dissemination

This trial was reviewed and given a favourable opinion by the Wales REC 7 Ethics Committee (reference: 23/WA/0085). Each participating centre is required to provide evidence of local confirmation of capacity and capability prior to starting recruitment to the trial. Participants have the right to withdraw from the trial at any time; they can also choose to stop completing questionnaires but remain in the trial for routine data collection only. Participants who choose to withdraw will be treated according to their hospitals’ standard procedures. Data obtained up until a patient’s withdrawal will be retained and used for analysis, unless the patient is enrolled under the waiver of consent and then, on regaining capacity, does not give permission for this to be used.

A plain English summary of the trial results will be prepared and disseminated with the help of our patient advisory group, including via social media, and provided to trial participants via post or email according to their preference. We will report our results via a high-impact medical journal and present findings at international medical, respiratory and emergency conferences. The results will be of high clinical importance, and as the trial uses techniques already used in other types of pneumothoraces, we anticipate rapid translation of results into routine clinical management of SSP and incorporation into national and international guidelines. The results will be posted on the publicly available registry (ISRCTN). A summary of the results will be submitted to the Health Research Authority (HRA) within 12 months of the end of the study in line with HRA guidelines.
