# Supplementary material for: Pragmatic non-inferiority Randomised trial Investigating Needle aspiration versus ChEst drain for Secondary Spontaneous Pneumothorax (the PRINCE-SSP study): study protocol for a randomised non-inferiority trial
Source: BMJ Open. 2024 Dec 26;14(12):e093700. doi: 10.1136/bmjopen-2024-093700 (PMC11683926; doi:10.1136/bmjopen-2024-093700)
Supplement: Supplementary file 1 [file bmjopen-2024-093700.reviewer_comments.pdf]

PEER REVIEW HISTORY

BMJ Open publishes all reviews undertaken for accepted manuscripts. Reviewers are asked to complete a checklist review form and are provided with free text boxes to elaborate on their assessment. These free text comments are reproduced below.

ARTICLE DETAILS

Title (Provisional)

Pragmatic non-inferiority Randomised trial Investigating Needle aspiration versus ChEst drain for Secondary Spontaneous Pneumothorax (The PRINCE-SSP study): a study protocol for a randomised non-inferiority trial

Authors

Walker, Steven; Taylor, Ellie; McAndrew, Amy; Carlton, Edward; Chaudhuri, Ella; Ghorpade, Pooja; Goodwin, Charlotte; Connors, James; Cook, Heather; Luengo-Fernandez, Ramon; Creanor, Siobhan; Maskell, Nick A

This article was not externally reviewed at BMJ Open. Protocols that have been independently assessed prior to submission to BMJ Open are usually fast-tracked to publication on the grounds that further substantial changes will not be possible. This independent assessment will usually be external, independent review for both a substantial grant award from a non-commercial or government funder and ethics approval.
